# Factors Affecting Concentration and Attentiveness in the Classroom of a Medical College in South India

**DOI:** 10.7759/cureus.86028

**Published:** 2025-06-14

**Authors:** Rajeswari I, Meena T S, Vidhya Selvam, Pavithra M

**Affiliations:** 1 Obstetrics and Gynaecology, Sree Balaji Medical College and Hospital, Chennai, IND

**Keywords:** attentiveness, clinical communication training, effective concentration, learning modalities, medical education

## Abstract

Objective: Attentiveness and concentration are critical for effective learning, particularly in demanding fields such as medical education. This study aimed to investigate various internal and external factors influencing medical students’ focus during classroom sessions.

Methods: A cross-sectional survey was conducted among 300 medical students (150 males and 150 females) using a structured questionnaire addressing subject-related, student-oriented, and learning mode factors affecting attention and concentration. Responses were analyzed for frequency, percentages, and gender-based differences using chi-square tests.

Results: Findings revealed that internal factors such as personal interest in the subject and emotional stability significantly impacted student attentiveness. Physiological factors, including prior meal status, also influenced concentration, especially during post-lunch sessions. External factors, such as teaching methods incorporating audiovisual aids and interactive techniques, were associated with improved focus. Environmental elements like noise pollution, lighting, and ventilation further affected concentration levels. The attentiveness of peers emerged as a novel factor influencing individual focus. No statistically significant differences were observed between male and female students across the measured variables (p > 0.05).

Conclusions: Medical students’ attentiveness is shaped by a complex interaction of intrinsic motivation, psychological well-being, instructional quality, social context, and physical learning environments. To enhance concentration and academic performance, educators should adopt diverse, student-centered teaching strategies and ensure conducive classroom conditions. Institutional support aimed at promoting student well-being is also essential. Future research should employ longitudinal and mixed-method designs to further explore these multifaceted influences and develop effective interventions.

## Introduction

Concentration and attentiveness are among the most important components of effective learning, particularly in the demanding environment of medical education. These cognitive functions are essential for acquiring both theoretical knowledge and practical clinical skills, which are critical for future medical practice. In the high-stakes context of medical training, even minor lapses in focus can result in knowledge gaps that may compromise professional competence and patient care. The importance of concentration and attentiveness has gained increasing attention from educators and academic planners, who now recognize that learning outcomes in medical institutions are not determined solely by curriculum content, but also by how that content is delivered and received by students.

Numerous psychological, physiological, and environmental factors influence a student's ability to concentrate. Psychological factors include motivation, emotional state, mental fatigue, and stress levels. Physiological variables such as hunger, sleep quality, and general health status also play a major role in determining cognitive performance during lectures or study sessions. Environmental factors, including the classroom setting, lighting, acoustics, and peer behavior, can further influence students’ focus and engagement. In recent years, there has been a strong emphasis on optimizing these variables to improve the quality of education in medical schools [1,2].

The modern medical curriculum is rigorous and content-heavy, demanding sustained attention and mental stamina from students. In such a learning environment, attentiveness is crucial not only for academic achievement but also for professional development. Students are expected to internalize complex biomedical knowledge, apply it in clinical scenarios, and demonstrate critical thinking, all of which require a high level of sustained focus. However, students often struggle with attentiveness due to multiple overlapping stressors, including exam pressure, clinical duties, and time constraints. These challenges can impair information processing, retention, and overall academic performance [1,2].

Mastery of the subject and individual topics has also been shown to play an important role in maintaining attentiveness in the classroom. Students who have a strong foundational understanding of a topic or possess a keen interest in a particular subject are more likely to remain engaged during lectures. In contrast, unfamiliarity or lack of interest may lead to cognitive disengagement [3,4]. Several studies have demonstrated that concentration can be enhanced by altering influencing variables such as teaching style, the degree of interactivity between instructors and students, and the physical organization of the learning environment [5,6].

In addition, personal factors such as intrinsic motivation, peer interaction, and emotional well-being significantly influence classroom concentration. External distractions such as noise, classroom overcrowding, and even post-meal drowsiness have been reported to reduce attentiveness among students [7,8]. It is also important to acknowledge that cognitive performance is dynamic and context-dependent. Factors like time of day, mental fatigue, and variations in teaching methodology can result in fluctuations in students’ ability to maintain focus during different sessions [9,10].

At this medical college, where a diverse group of final-year medical students attend theoretical lectures across various disciplines, understanding the specific factors affecting their concentration and attentiveness becomes highly relevant. Final-year students are often under significant academic and clinical pressure, making it critical to identify barriers to effective learning. This study aims to explore students’ perspectives regarding the most influential factors that impact their classroom concentration and attentiveness. By identifying these determinants, educational planners and faculty can develop targeted interventions to foster better engagement, enhance the learning environment, and ultimately improve academic and clinical outcomes.

## Materials and methods

Study design and setting

This study was designed as a cross-sectional, questionnaire-based analysis aimed at assessing the various factors that influence concentration and attentiveness among final-year medical students during their academic sessions. The cross-sectional design was chosen for its feasibility and effectiveness in identifying associations between individual, environmental, and institutional factors and cognitive focus at a specific point in time. Given the intensive academic load and high expectations placed on final-year medical students, this cohort provides a valuable lens through which to examine both intrinsic and extrinsic factors affecting student engagement.

The study was conducted at a tertiary care teaching hospital affiliated with a government medical college in Chennai, Tamil Nadu, India. This setting was deemed suitable for the study as it hosts a diverse and competitive cohort of undergraduate medical students, representing a cross-section of varied socioeconomic, cultural, and educational backgrounds. These students are regularly exposed to a demanding curriculum that includes both theoretical classroom-based learning and practical clinical training. The environment, therefore, presents an ideal backdrop to investigate the cognitive and behavioral dynamics that may affect classroom attentiveness and overall academic performance. The anticipated duration of the study was three months, allowing adequate time for participant recruitment, data collection, validation, and analysis.

Study population and inclusion criteria

The target population comprised final-year medical students enrolled at the institution during the study period. This academic year was specifically chosen as it is known for its rigorous academic schedule, extensive clinical postings, and preparation for final professional examinations, all of which demand high levels of concentration, mental stamina, and time management. As such, these students face multifaceted stressors, making them an appropriate group for evaluating factors that impact attentiveness in classroom settings.

The inclusion criteria required participants to be actively enrolled in the final year of their medical program, present during the data collection phase, and willing to provide informed consent for participation. Students who were absent during the survey period or who declined to participate were excluded from the study. Ethical approval was obtained from the Institutional Human Ethics Committee of Sree Balaji Medical College and Hospital (Chennai, TN, IND) before data collection (approval no. 002/SBMCH/IHEC/2025/2396). All ethical guidelines pertaining to human subjects research were strictly followed.

Sample size calculation

The sample size was determined based on the formula used for estimating a population mean with relative precision. Drawing from existing literature and a prior study on a similar topic [11], the mean score for concentration was estimated to be 4.29, with a standard deviation of 0.91. A relative precision of 1% and a confidence level of 95% were used as statistical parameters to calculate the minimum required sample size. Based on these values, the sample size was computed to be 256 students. Thus, the sample size was rounded to 300 students to ensure robust representation, account for potential non-response or incomplete data, and increase statistical power. This number was deemed appropriate for conducting subgroup analyses and ensuring the generalizability of the results to a broader population of medical students.

Sampling methodology and data collection

A universal sampling strategy was employed, wherein all eligible final-year medical students (n = 300) who met the inclusion criteria were invited to participate in the study. This approach was preferred to ensure inclusiveness and minimize sampling bias, particularly since the final-year batch size at the institution closely aligned with the required sample size. Participation was voluntary, and informed consent was obtained from each student prior to their involvement in the study.

Data were collected using a structured, self-administered questionnaire, specifically developed for this study (Appendix A). The questionnaire was designed after reviewing existing tools and literature related to classroom attention, cognitive load, and academic performance [11,12]. It included closed-ended items aimed at capturing information across multiple domains believed to influence concentration and attentiveness. This questionnaire was designed to assess various factors influencing students' attention and learning during classroom sessions. It includes 11 questions grouped into three categories: subject-related factors, student-related factors, and learning mode factors. The subject-related section explores students’ clinical orientation, prior knowledge, and perceived sufficiency of study material. The student-related section examines personal interest in the subject, the impact of meal status, peer distractions, and emotional background on concentration. The learning mode section evaluates the effectiveness of audiovisual aids, teaching methods, and environmental conditions such as noise, lighting, and ventilation. Responses were recorded as 'yes' or 'no' from a total of 300 students, providing both frequency and percentage to analyze the prevalence of each factor affecting classroom attention and engagement.

As per the ethics committee’s guidance, the questionnaire was carefully developed to focus on the most relevant and practical factors influencing concentration and attentiveness among medical students, ensuring minimal respondent burden and maximum clarity. The 11-item tool, created by authors Vidhya and Rajeswari, was structured into three domains, namely subject-related factors (three items), student-oriented factors (four items), learning mode (two items), and environmental factors (two items). A thorough literature review informed item generation, and content validity was established through expert review by five medical education specialists, with all items achieving an item-level content validity index (CVI) of ≥0.80. The tool was then pilot tested among 30 final-year medical students for clarity and comprehension, and minor wording refinements were made accordingly. Internal consistency was verified with a Cronbach’s alpha of 0.78, indicating good reliability. For more reliability analysis, internal consistency was examined using the Kuder-Richardson formula 21 (KR-21), yielding a coefficient of 0.78, indicating acceptable reliability for dichotomous item responses.

The ethics committee emphasised the importance of focusing on a concise and context-specific tool tailored to the local medical education setting, rather than adopting more comprehensive but potentially burdensome instruments used in prior studies. Therefore, the number of items was intentionally limited to reduce respondent fatigue while still capturing the key domains influencing student attentiveness. The administration of the questionnaire was done during scheduled lecture hours, under the supervision of the research team, to ensure a standardized and neutral data collection environment. Respondents were assured of the confidentiality and anonymity of their responses, and they were encouraged to answer honestly without any fear of academic repercussions.

Data analysis

All collected data were coded and entered into SPSS Statistics version 28.0 (IBM Corp., Armonk, NY, USA) for statistical analysis. Descriptive statistics were used to summarize the data. Categorical variables (gender, hostel residency, and self-reported use of digital devices) were summarized using frequencies and percentages, while continuous variables (concentration scores) were expressed as means with standard deviations or medians with interquartile ranges, depending on the distribution assessed using the Shapiro-Wilk test.

To examine associations between different factors and concentration scores, inferential statistical tests were applied. For comparing proportions between groups, the chi-square test or Fisher’s exact test (for expected frequencies <5) was used. A p-value < 0.05 was considered statistically significant for all analyses.

## Results

A total of 300 students participated in the questionnaire survey conducted by the principal investigator. The respondents included an equal number of male and female students, with 150 (50%) males and 150 (50%) females. Each participant responded to questions related to subject understanding, personal and emotional factors, and learning environment. The balanced gender distribution allows for a comparative analysis of responses between male and female students across various influencing factors.

Table 1 presents the responses of 300 students to a set of 11 questions assessing factors that influence classroom attention and learning. For the question on clinical orientation to the topic before class, 215 students (71.7%) responded 'yes' while 85 (28.3%) said 'no.' Prior knowledge of the subject was reported by 188 students (62.7%), with 112 (37.3%) reporting a lack of it. A total of 219 students (73.0%) found the volume of material sufficient. Personal interest in the subject was expressed by 213 students (71.0%). Regarding physiological factors, 202.5 students (67.5%) felt their meal status affected attention post-lunch. Peer distraction was acknowledged by 214 students (71.5%), and 237 (79.0%) agreed that emotional background impacted concentration. Audiovisual aids were found beneficial by 251 students (83.7%), and 218 (72.7%) felt teaching methods enhanced attention. Environmental factors also played a role: 210 students (70.0%) were affected by noise pollution, and 252 (84.0%) reported that lighting and ventilation influenced their classroom concentration. These results highlight the multifactorial nature of attention and learning in academic settings.

**Table 1:** Response to the study questionnaire among the study population (n=300) Questionnaire created by authors Vidhya and Rajeswari.

No.	Question	Yes: Frequency (%)	No: Frequency (%)
1	Are you clinically oriented to the topic before taking class?	215 (71.7%)	85 (28.3%)
2	Do you have prior knowledge about the subject?	188 (62.7%)	112 (37.3%)
3	Is the volume of material sufficient?	219 (73.0%)	81 (27.0%)
4	Do you have personal interest in the subject?	213 (71.0%)	87 (29.0%)
5	Does prior meal status affect your attention in post-lunch sessions?	202 (67.5%)	97 (32.5%)
6	Does the attention of friends disturb your concentration?	214 (71.5%)	85 (28.5%)
7	Does your emotional background disturb your concentration?	237 (79.0%)	63 (21.0%)
8	Is using audiovisual aids beneficial to you?	251 (83.7%)	49 (16.3%)
9	Does the teaching method draw your attention?	218 (72.7%)	82 (27.3%)
10	Does noise pollution disturb your attention?	210 (70.0%)	90 (30.0%)
11	Does proper lighting/ventilation disturb your concentration in the classroom?	252 (84.0%)	48 (16.0%)

Table 2 shows the gender-wise distribution and row-wise percentage of students who responded 'yes' to three subject-related factors, with each row total considered as 100%. Out of the 219 students who felt the volume of material was sufficient, 105 (47.9%) were males and 114 (52.1%) were females. Among the 215 students who felt clinically oriented to the topic before class, 102 (47.4%) were males and 113 (52.6%) were females. For prior knowledge of the subject, 90 out of 188 (47.9%) were males and 98 (52.1%) were females. The chi-square values and p-value indicate no statistically significant difference between male and female students in their responses to these subject-related questions.

**Table 2:** Gender-wise distribution of responses to subject-related factors among students A p-value <0.05 is considered statistically significant.

Subject-related factors	Male	Female	Total (n)	Chi-square	p-value
Volume of material (yes)	105 (47.9%)	114 (52.1%)	219	0.0127	0.997
Clinically oriented topic (yes)	102 (47.4%)	113 (52.6%)	215	0.035	0.813
Prior knowledge (yes)	90 (47.9%)	98 (52.1%)	188	0.72	0.354

Table 3 illustrates the distribution of responses from male and female students regarding student-oriented factors affecting their learning and attention. Among the 213 students expressing personal interest in the subject, 105 (49.3%) were males and 108 (50.7%) were females. For the impact of prior meal status on attention, 203 students responded positively, with 97 (48.0%) males and 105 (52.0%) females. Regarding whether friends’ attentiveness disturbs concentration, 117 males (54.4%) and 97 females (45.6%) agreed out of 215 total respondents. Lastly, 237 (105 males (44.3%) and 132 females (55.7%) students reported emotional background disturbances. The p-values indicate no statistically significant difference between genders in these responses.

**Table 3:** Gender-wise distribution of responses to student-oriented factors affecting learning and attention A p-value <0.05 is considered statistically significant.

Student-oriented factors	Male	Female	Total (n)	Chi-square	p-value
Personal interest in subject (yes)	105 (49.3%)	108 (50.7%)	213	0.667	0.191
Prior meal status affecting attention (yes)	97 (48.0%)	105 (52.0%)	203	1.76	0.234
Attentiveness of friends disturbing concentration (yes)	117 (54.4%)	97 (45.6%)	215	4.12	0.098
Emotional background disturbing concentration (yes)	105 (44.3%)	132 (55.7%)	237	3.78	0.073

Table 4 summarizes the responses of male and female students regarding learning mode and environmental factors that affect their classroom attention. Among the 251 students who found using audiovisual aids beneficial, 125 (49.8%) were males and 126 (50.2%) females. For teaching methods that promote student engagement, 108 males (49.5%) and 110 females (50.5%) responded positively out of 218 students. Regarding noise pollution disturbance, 102 males (48.6%) and 108 females (51.4%) agreed from a total of 210 respondents. In terms of the impact of proper lighting and ventilation, 252 students responded affirmatively, with 111 males (44.0%) and 141 females (56.0%). The chi-square test indicates no statistically significant difference between male and female students in these factors.

**Table 4:** Gender-wise distribution of responses to learning mode and environmental factors influencing classroom attention A p-value <0.05 is considered statistically significant.

Learning mode factors	Male	Female	Total (n)	Chi-square	p-value
Using audiovisual (yes)	125 (49.8%)	126 (50.2%)	251	0.1352	0.549
Teaching method (yes)	108 (49.5%)	110 (50.5%)	218	0.45	0.656
Environmental Factors					
Noise pollution (yes)	102 (48.6%)	108 (51.4%)	210	1.87	0.215
Proper lighting and ventilation (yes)	111 (44.0%)	141 (56.0%)	252	3.99	0.075

## Discussion

This study explored a range of factors influencing attentiveness and concentration among medical students, providing valuable insights into both intrinsic and extrinsic determinants that affect academic performance and overall well-being. Attention and concentration are crucial for effective learning, especially in medical education, where cognitive load is high and the material is complex. The findings align with and expand upon existing literature, emphasizing the multifactorial nature of student focus, involving personal, instructional, social, and environmental dimensions.

Internal factors

Internal factors primarily relate to individual characteristics and psychological traits. Our results highlight that personal interest and intrinsic motivation significantly enhance attentiveness, consistent with prior findings from Fasihi et al. [5], which show that student engagement increases with active involvement and interest in the subject. These findings also corroborate earlier research Raca et al. [13], which identified motivation and interest as key drivers of concentration, particularly among male students. Psychological factors such as emotional stability and stress management also play pivotal roles. Emotional background was found to notably affect concentration, especially among female students, aligning with findings from Haresabadi and Raofian [14] and supporting the critical role of emotional regulation in sustaining focus.

Furthermore, physiological factors like prior meal status influenced attentiveness in post-lunch sessions, echoing the work of Hershner and Chervin [4], who emphasized how sleep quality and post-meal drowsiness impact cognitive capacity. These physiological considerations underscore the importance of maintaining physical well-being alongside psychological preparedness to optimize learning outcomes. Cognitive resilience and coping mechanisms, while not directly measured, are implicitly linked to improved attention and stress mitigation, as described in broader educational research by Tabarsa et al. [15].

External factors

This study further examines external or environmental influences, including classroom dynamics, teaching methods, and physical settings. Interactive and multimedia teaching methods, such as audiovisual aids, were strongly associated with improved concentration. Supporting evidence from Rahiminia et al. [11] demonstrates that active participation and the use of technology enhance student engagement. These findings reinforce calls for educational institutions to incorporate diverse instructional techniques that cater to varied learning styles.

Environmental conditions like noise pollution, lighting, and ventilation also emerged as significant factors impacting attention, consistent with the observations of Naderi et al. and Aliabadi et al. [16,17], who stressed the importance of optimizing classroom acoustics and ergonomic conditions. Poor lighting or ventilation can distract students and reduce cognitive efficiency, highlighting the need for well-designed learning spaces that minimize such barriers.

Notably, the social environment, including the attentiveness of peers, surfaced as an influential yet less studied factor. Our results suggest that the focus level of friends in the classroom can either enhance or disrupt individual concentration. This peer influence on attentiveness is a novel contribution to the literature, as few prior studies have examined this social dimension of classroom dynamics [18,19]. It points to the importance of fostering a positive and focused classroom culture to support collective learning.

Professor-related factors also play a crucial role in sustaining student attention. As reported by Mangal [20], the mastery of content, teaching style, and interpersonal communication from educators significantly influence student motivation and engagement. Female students in particular value professors’ knowledge and emotional readiness, while male students emphasize motivation and interest in the topic, suggesting nuanced gender differences in factors affecting attention. These insights underscore the need for faculty development programs to enhance not only content delivery but also the affective and relational aspects of teaching.

Implications for education and practice

The findings underscore the complexity of attentiveness in academic settings, calling for multifaceted strategies to foster effective learning. Educators should design curricula that integrate interactive, student-centered teaching methods and leverage audiovisual technologies to sustain interest and participation. Institutions must prioritize creating optimal physical environments, addressing noise control, lighting, and ventilation, to reduce distractions and enhance focus.

Moreover, recognizing the role of psychological and physiological factors, educational support services should emphasize student well-being through stress management resources, mental health counseling, and awareness about the impact of nutrition and sleep on cognitive function. Encouraging positive peer dynamics and attentiveness within classrooms can further augment learning environments.

The gender-based differences in attention-related factors highlight the importance of tailored approaches that consider diverse student needs and preferences. Faculty training should incorporate strategies for emotionally responsive teaching and inclusive engagement to address these variations effectively.

Limitations and future directions

While this study offers comprehensive insights, it is limited by its cross-sectional design, which precludes causal inference. Longitudinal research is needed to understand how attentiveness fluctuates over time and across different academic contexts. The study’s focus on undergraduate medical students from a single institution limits the generalizability of findings to other populations or educational settings.

Future studies should explore additional variables, such as digital distractions, sleep patterns, and socioeconomic factors, using mixed-method approaches to capture the complexity of attentiveness. Investigating interventions that enhance internal motivation and improve learning environments could provide practical solutions to optimize student focus.

## Conclusions

This study highlights that medical students’ attentiveness and concentration are influenced by a complex interplay of internal factors such as personal interest, emotional stability, and physiological states, as well as external factors including teaching methods, classroom environment, and peer dynamics. No significant gender differences were observed in these determinants, suggesting that interventions to enhance focus should be inclusive and multifaceted. Educators and institutions must prioritize creating engaging, supportive, and well-designed learning environments while also addressing students’ psychological and physical well-being. Future efforts should focus on developing targeted strategies to foster sustained attention and improve academic outcomes among medical students.

## Appendices

Appendix A 

Table 5 showcases the questionnaire used for assessing factors affecting the concentration and attentiveness of medical students in classroom settings.

**Table 5:** Questionnaire Questionnaire created by authors Vidhya and Rajeswari.

Category	Question	Response options
Subject-related factors	Are you clinically oriented to the topic before taking the class?	Yes / No
	Do you have prior knowledge about the subject?	Yes / No
	Is the volume of material sufficient?	Yes / No
Student-related factors	Do you have a personal interest in the subject?	Yes / No
	Does prior meal status affect your attention in post-lunch sessions?	Yes / No
	Does the attentiveness of your friends disturb your concentration?	Yes / No
	Does your emotional background disturb your concentration?	Yes / No
Learning mode factors	Is using audiovisual aids beneficial to you?	Yes / No
	Does the teaching method keep you attentive?	Yes / No
Environmental factors	Does noise pollution disturb your attention?	Yes / No
	Does improper lighting or ventilation affect your concentration during class?	Yes / No
